# Building bridges, breaking silos: a social network analysis of collaboration in an implementation science center for addiction health services

**DOI:** 10.1093/tbm/ibag074

**Published:** 2026-10-07

**Authors:** Helene Chokron Garneau, Wouter Vermeer, Savanna Stromberg, Praise Olatunde, Mark McGovern

**Affiliations:** Center for Dissemination & Implementation at Stanford (C-DIAS), Palo Alto, CA, United States; Stanford University School of Medicine, Palo Alto, CA, United States; Center for Dissemination & Implementation at Stanford (C-DIAS), Palo Alto, CA, United States; Northwestern University Feinberg School of Medicine, Chicago, IL, United States; Center for Dissemination & Implementation at Stanford (C-DIAS), Palo Alto, CA, United States; Stanford University School of Medicine, Palo Alto, CA, United States; Center for Dissemination & Implementation at Stanford (C-DIAS), Palo Alto, CA, United States; Stanford University School of Medicine, Palo Alto, CA, United States; Center for Dissemination & Implementation at Stanford (C-DIAS), Palo Alto, CA, United States; Stanford University School of Medicine, Palo Alto, CA, United States

**Keywords:** social network analysis, addiction health services, implementation science, center

## Abstract

**Background:**

Large scale collaborative or research centers are designed to accelerate scientific progress, promote sustained collaboration, and bridge gaps across areas of expertise. Traditionally, metrics of evaluation for such centers have focused on quantifiable outcomes, such as number and impact of publications. These take time to materialize yet formation, growth, and evolution of collaborative ties, which are fundamental to achieve these quantifiable outcomes, are seldom examined.

**Purpose:**

To investigate collaborative ties, we applied social network analysis to the NIH-funded Center for Dissemination & Implementation at Stanford (C-DIAS). We illustrate the development and structure of collaborative, cross-disciplinary, and mentorship relationships within the center during its first three years.

**Methods:**

Data was collected from C-DIAS members in 2020, 2023, and 2025. Social network analyses examined four components: growth of interaction and collaboration networks, bridging disciplinary boundaries, establishing national reach, and building capacity through mentorship.

**Results:**

Across three waves, C-DIAS expanded cross-disciplinary networks, with boundary-spanning ties growing from 21 to 180 while maintaining high connectivity. Interaction and collaboration networks grew substantially, converting interpersonal contact into productive partnerships. Geographic reach increased from 10 to 28 institutions, and near-universal mentorship integrated new members, supporting sustained development of early-career investigators across cohorts.

**Conclusions:**

In 3 years, C-DIAS cultivated a highly connected, disciplinary network linking addiction health services and implementation science researchers. Intentional infrastructure fostered collaboration, mentorship, and national reach, demonstrating that structured networks can break silos and accelerate the translation of research into practice.

## Teaser

A three-year social network study reveals how an NIH-funded research center successfully brought together researchers with different areas of expertise to form a highly connected national network. Findings demonstrate that intentional relationship-building infrastructure can break down silos and accelerate the translation of addiction health services research into real-world practice.

Implications **Practice:** Federally funded coordinating and research centers should measure synergy and collaboration across projects and people to ensure and enhance collaborative capacity and facilitate the translation of research findings into practice.

**Policy:** Research and collaborations within a center can drive field-wide change and population health impact in ways that individual projects cannot, producing findings that are more scalable and sustainable than those from isolated studies.

**Research:** Comparing different center models on their ability to achieve synergistic goals can help the field understand how to improve center functioning and maximize scientific and public health impact.

## Introduction

Science and research projects are typically conducted independently. This entrepreneurialism indeed has countless merits, yet there are often missed opportunities for synergy, rapid scientific innovation, and resulting accelerated progress and impact. Leveraging knowledge across disciplinary boundaries may be an alternative approach to create a total outcome that is greater-than-the-sum-of-its-parts. However, the coordination efforts required are often perceived as a barrier. To foster interdisciplinary research, the National Institutes of Health (NIH) have made substantial investments in large scale collaborative research mechanisms such as Program Project Grants (P series) and Cooperative Agreements (U series) [1]. These funding mechanisms are meant to support centers that build bridges, accelerate scientific progress, and facilitate more rapid translation of evidence-based treatments into widespread use. Their intent is to bring together a nucleus of experts across disciplines over a five-year period to: address research problems that are too complex for an individual research team; build capacity by training a new generation of boundary spanning researchers; and enable data harmonization or standardization to multiply the impact of findings [2, 3].

The science of team science shows that research centers succeed largely through the collaborations they build, not through individual effort alone: cross-disciplinary teams produce advances that separate investigators cannot [4, 5]. A collaboration network is a direct, visible representation of those relationships, which makes it a natural way to evaluate whether a center is working. Implementation science points to the same conclusion from a different angle. Translating research into practice depends on the connections among researchers, practitioners, and decision-makers; widely used frameworks treat these networks as a determinant of implementation success [6], and research on network interventions shows that the structure of connections can be used to speed up the spread of new practices [7]. Together, these fields establish that a center’s collaborative relationships are the mechanism through which it accelerates translation and therefore a meaningful target for evaluation.

Evaluation criteria for research or coordinating centers remain poorly specified. Objective measurement and analysis of center programs’ performance, especially of impact, are difficult. The focus tends to be on quantifiable outcomes such as quality and impact of publications, effectiveness in facilitating collaborative studies, and success in attracting new investigators to the field. These metrics, however, provide limited insight into the collaborative processes that centers are specifically designed to foster and take time to materialize. We posit that social network analysis (SNA) offers a powerful complementary approach to evaluating centers’ ability to develop collaboration processes by quantifying the structure, density, and evolution of collaborative relationships [8]. By mapping patterns of interaction, collaboration, mentorship, and clustering across disciplinary boundaries, SNA can reveal whether centers are successfully breaking down silos, assimilating new members, and building the sustained relationships necessary for transformative interdisciplinary work, and provides such insights on a near real-time basis.

The addiction health services field exemplifies both the urgent need for interdisciplinary collaboration and the challenges inherent in achieving it [9–11]. Despite substantial advances in understanding addiction and development of evidence-based interventions including medications for opioid use disorder and behavioral therapies, implementation of these treatments remains disappointingly limited [11–13]. Closing this research-to-practice gap requires integration of expertise spanning substance use treatment, implementation science, organization and financing of services, and advanced quantitative methods; areas of expertise that have traditionally operated independently. Recognizing this need, in 2022 the National Institute on Drug Abuse funded the Center for Dissemination & Implementation at Stanford (C-DIAS; P50DA054072) through the center grant mechanism (PAR-19-259). C-DIAS represents the first NIH-funded center dedicated to implementation science for addiction health services, with the explicit goal of improving access to evidence-based addiction health services while simultaneously advancing implementation research methodology. C-DIAS (Fig. 1) comprises an Administrative Core, a Research Core, and three Research Projects [14]. The Administrative Core provides the center’s operational infrastructure and leads communications, dissemination, and educational programming, including the C-DIAS Fellowship in Addiction Implementation Science. The Research Core is organized into three integrated sections; Methods & Measures, Design & Modeling, and Organization & Financing of Services, each comprising 6–8 C-DIAS faculty who contribute methodological expertise across the center’s three Research Projects and develop resources for the field at large. The Research Projects are linked to C-DIAS through shared members and resources, with some Research Core members serving as project co-investigators and project leads serving on the Research Core. Each project and section hosts 1–2 fellows on a yearly basis. As embedded section or project members, fellows have opportunities to lead scholarly contributions.

**Figure 1:**
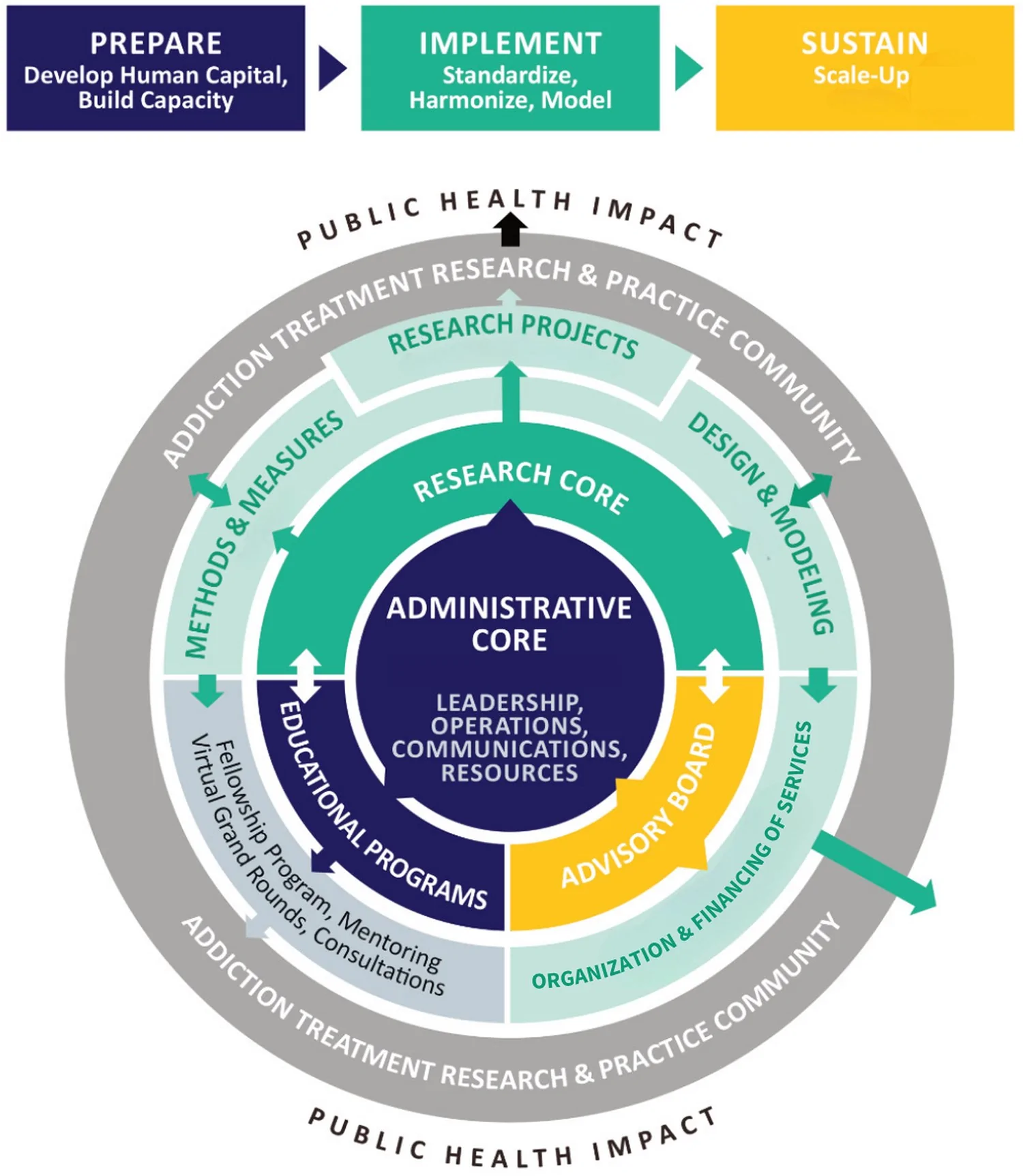
C-DIAS overall center.

Central to the C-DIAS model is the hypothesis that sustained interaction among researchers with complementary expertise in substance use treatment, implementation research, methods and measures, design and modeling, and organization and financing of services would catalyze innovation at disciplinary intersections, accelerate the pace of discovery-to-implementation, and build capacity for the next generation of addiction-focused implementation scientists.

We employed SNA to map C-DIAS’ network formation and evolution during its first three years of operation and specifically examined: (i) how interaction and collaboration networks have grown and evolved in terms of size, density, and structural properties; (ii) the extent to which the center has successfully bridged disciplines of addiction health services, implementation science, and organization and financing of services; (iii) whether C-DIAS has established geographically distributed collaborations; and (iv) how mentorship relationships have developed to support capacity building, particularly for early-career investigators entering the field.

## Methods

### Participants and data collection

C-DIAS membership grew from 25 members at the time of funding in 2022 to 49 in 2025. Of the 49 members in 2025, 28 were C-DIAS faculty and 21 were C-DIAS fellows. Faculty were recruited by C-DIAS leadership, and fellows were selected by the Educational Programs committee. Thirty-nine members (80%) held a PhD and 10 (20%) held an MD. Approximately half were at the Assistant or Associate Professor level and half at the Professor level.

Consistent with reporting guidelines for social networks in health research [15], we specify the network boundary explicitly: this is a complete-network study in which the network at each wave comprised all C-DIAS faculty and fellows formally affiliated with the center at the time of that wave’s survey. Nodes represent individual center members. Interaction and collaboration ties are undirected, whereas mentorship ties are directed.

Data used to evaluate the impact of C-DIAS were obtained to capture the evolution of the breadth and density of the C-DIAS relationships, network, and collaborative ties. The survey was distributed to C-DIAS faculty, affiliates, and fellows. Data collection occurred in three waves: Wave 1 (2020) was collected during the grant development period, prior to the initial submission for funding (*N* = 25), Wave 2 (2023) was collected a little over one year into the 5 year funding period (*N* = 40), and Wave 3 (2025) was collected in the third grant year (*N* = 49).

### Measures

Three types of information were assessed: (i) frequency of contact (annually, quarterly, monthly, weekly); (ii) collaboration ties on activities such as developing new research, submitting grants, presenting research results, publishing a paper, information brainstorming, collegial support, and serving on a center, committee, or institute; and (iii) mentoring relationships. Sample questions are “Please indicate how frequently you have been or are in contact with [name]”; “Please select all of the collaboration activities you have engaged in with the following people since [date]”; “Please indicate which individuals have either mentored you or been mentored by you since [date]”. For the mentoring item, respondents then selected, for each named individual, the direction of the relationship: “I mentored them,” “They mentored me,” “Bi-directional mentoring,” or “Neither” allowing mentorship ties to be represented as directed edges.

### Analysis

Analyses were conducted across two platforms: R version 4.5.2 was used for data preparation and cleaning, and Gephi version 0.10.1 was used for network metric calculation and visualization. The resulting analysis was divided into four components further described below.

### Growth of interaction and collaboration networks

To evaluate network growth, we tracked the evolution of two distinct but related network types across all three waves of data collection. Interaction networks captured the frequency of contact between individuals (annually, quarterly, monthly, or weekly), while collaboration networks captured engagement in joint research activities including developing new research projects, submitting grant applications, and co-authoring publications. Interaction represents a necessary foundation for collaboration but does not guarantee it. Effective networks balance sufficient density to enable knowledge sharing with enough structural diversity to bring in novel perspectives [16, 17]. However, no single metric comprehensively captures network health, and optimal values are highly context specific [8]. We therefore employed a complementary suite of measures that together characterize network connectivity, span, and clustering.

#### Connectivity

Degree, the number of connections an individual maintains, illustrates the heterogeneity of engagement within the network. Examining degree distributions identifies isolates (individuals with no connections) and hubs (highly connected individuals who may bridge disciplinary or institutional boundaries). Network density, the proportion of all possible ties that are present, provides a complementary global measure of overall connectivity [2].

#### Span

Path length measures the number of steps required to connect two individuals through their network ties. Average path length indicates overall cohesiveness: shorter paths enable more efficient information flow and increase the likelihood of cross-pollination between distant parts of the network. Network diameter, the longest shortest path between any two members, reveals the breadth of the network and can identify peripheral subgroups that may be poorly integrated.

#### Clustering

At the micro-level, the clustering coefficient measures triadic closure: the proportion of an individual’s contacts who are also connected to each other. High clustering creates dense local neighborhoods that facilitate trust-building and knowledge exchange. At the macro-level, community structures can be detected as groups of individuals (communities) that are more connected internally than to those outside the cluster. We use modularity to quantify relative fit of the community structures within the network [3, 8]. Low modularity indicates a relatively weak community structure and thus relatively homogeneously integrated network, while high modularity suggests stronger fragmentation into distinct clusters. Modularity is therefore interpreted alongside density and path-length metrics rather than as a standalone index of integration.

### Bridging disciplinary boundaries

A central goal of C-DIAS is breaking down disciplinary silos. To evaluate progress toward this goal, we coded each network member’s primary disciplinary expertise in four domains aligned with C-DIAS’s organizational structure: implementation science, addiction health services, methods and models, and organization and financing of services. Members could be coded as experts in multiple domains if their work substantively spanned disciplines. Using these disciplinary classifications, we categorized each tie in the interaction and collaboration networks as either within-discipline (connecting two individuals with the same primary expertise) or cross-discipline (bridging different domains). We calculated the proportion of cross-disciplinary ties at each wave and compared these rates both to within-discipline connection rates and to expected values if ties formed randomly without regard to disciplinary boundaries. Increasing cross-disciplinary connectivity over time would indicate successful silo-breaking, while persistent clustering by discipline would suggest barriers to integration remain.

We also examined which specific discipline showed the strongest growth in cross-disciplinary connectivity, identifying potential “bridge disciplines” that may serve as natural connectors in the network and revealing persistent gaps where integration efforts should be intensified.

### Establishing national reach

C-DIAS is a geographically distributed network intended to serve as a national resource. To evaluate whether this vision materialized, we conducted geospatial network analysis by mapping the institutional locations of all network members at each wave. We geocoded each member’s home institution and overlaid interaction network ties onto a map of the United States for each wave of data, creating visual representations of the network’s geographic footprint over time.

### Building capacity through mentorship

Capacity building is fundamental to C-DIAS’s mission. To evaluate these efforts, we analyzed the formation and evolution of mentorship networks across all three waves. We tracked two distinct cohorts of C-DIAS fellows: cohort 1 entered prior to Wave 2 data collection, while cohort 2 entered prior to Wave 3.

We also analyzed the overall mentorship network structure to identify whether mentorship was concentrated among a small number of highly active mentors (suggesting potential bottlenecks) or distributed more broadly (indicating systemic capacity-building). By comparing mentorship patterns to the broader collaboration networks, we assessed whether C-DIAS successfully converted mentorship into sustained collaborative relationships that persist beyond formal training periods.

## Results

Survey response rates were excellent with 100% participation in Wave 1 (*n* = 25), 90% in Wave 2 (*n* = 36 of 40 invited participants), and 100% in Wave 3 (*n* = 49). All networks were treated as undirected unless explicitly stated otherwise, with a tie recorded as present if mentioned by either party. In cases where respondents reported different interaction frequencies or collaboration intensities, we retained the higher value.

### Growth of interaction and collaboration networks

#### Interaction networks

While contact networks were designed to capture any frequency of contact (annually, quarterly, monthly, or weekly) networks based on such a wide range of frequencies capture familiarity more than sustained engagement or interaction. To examine the actual activity within C-DIAS, we therefore focused on “regular” ties, i.e. those that occur at least monthly.

Regular ties constitute approximately one-third of all interactions across waves and capture the nucleus of interaction within this center over time. These regular interaction networks, while less dense than the overall contact networks, remained well connected (Table 1, Fig. 2). The networks grew substantially over time: from 25 to 40 to 49 individuals, and from 66 to 134 to 296 ties across Waves 1, 2, and 3, and while the second wave showed a slight dip in connectivity with density decreasing, the clustering coefficient declining, and the average path length increasing, these metrics rebounded strongly in Wave 3, surpassing initial values. The remarkably low average path lengths and network diameters reflect the presence of and critical role of center leadership (squares in Fig. 2) and specifically the center PI, maintaining connections to all members. While this creates a “spoke-and-wheel” structure where individuals are rarely more than two steps apart, it also introduces measurement artifacts that artificially inflate clustering coefficients and compress path lengths beyond what would occur in more distributed network structures. Community structure calculations identified an increasing number of communities over time within these networks, but both visual inspection and moderate modularity scores indicated these were weak divisions with substantial cross-community connectivity. While community membership of individuals shifted over time, these clusters signal the emergence of project-centered working groups within C-DIAS. The moderate modularity values indicate that despite this internal differentiation, substantial cross-cluster connectivity persists. The low-to-moderate modularity values (0.30, 0.31, 0.24; Table 1) indicate that the detected communities are weakly separated, with substantial cross-cluster connectivity rather than distinct silos. Reachability, not modularity, evidences the center’s cohesion: short average path lengths (1.79–2.05) and a small diameter (3–4) kept members within a few steps of one another across all waves, with no wave fracturing into disconnected components. Taken together, these patterns suggest C-DIAS has successfully balanced domain and project-specific focus with network-wide integration, precisely the integrated structure the center was designed to create rather than isolated silos.

**Figure 2:**
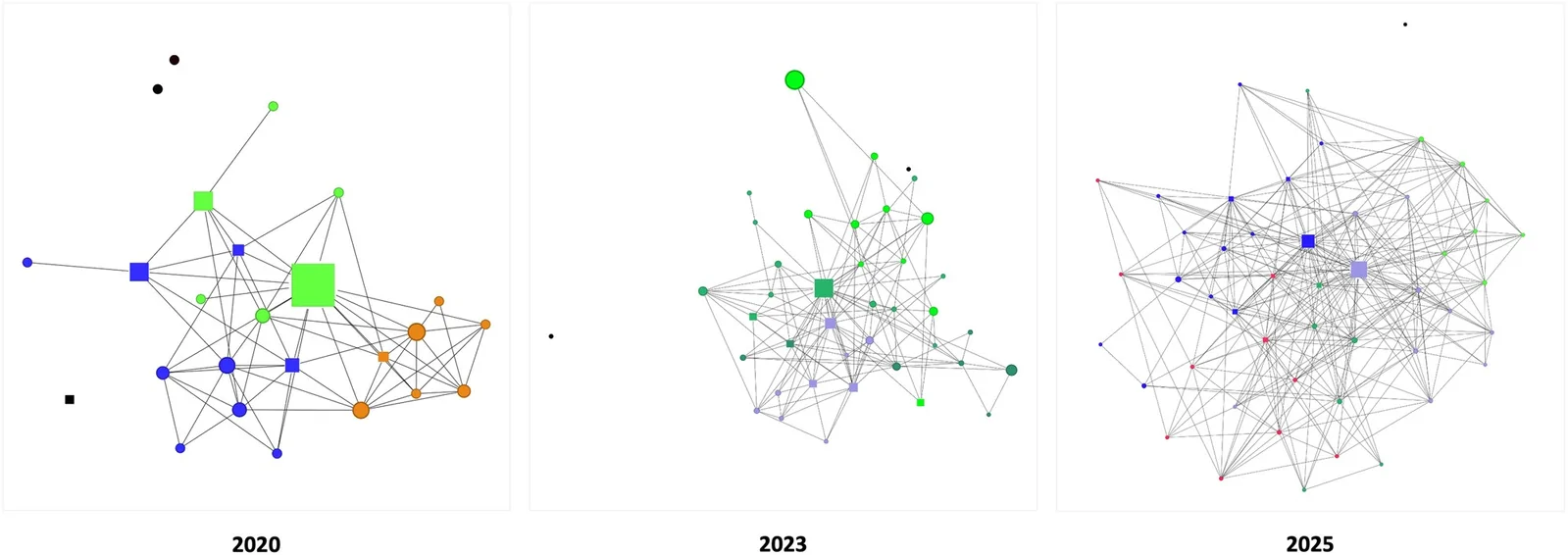
Trends in interaction that occur at least monthly (regular ties). Figure 2 displays the networks of interactions that occur at least monthly within the C-DIAS center for Wave 1 (left), Wave 2 (middle), and Wave 3 (right). Nodes represent individuals (squares highlight center leadership) and the lines between them indicate an interaction that occurs at least monthly. Node size reflects the relative degree, the number of ties of the individual. Node color in each of these panels captures the community structure of the nodes in that wave specifically.

**Table 1:** Network metrics for “regular” interaction networks (at least monthly) across waves.

	Wave 1	Wave 2	Wave 3
Number of individuals (*N*)	25	40	49
Number of ties (*E*) and (% of any contact)	66 (30.7%)	134 (29.26%)	296 (33.11%)
Number of ties of the most connected Individual (max degree)	15	26	39
Overall width of the network (diameter)	4	4	3
Average number of steps between individuals (average path length)	1.935	2.048	1.792
Proportion of total ties present (density)	22.0%	19.1%	25.2%
Percentage of neighbors that are connected (average clustering coefficient)	47.0%	53.5%	56.4%
Number of sub-structures (number of communities) [18]	3^a^	4^a^	5^a^
Isolates	3	2	1
Fit with community detection. Presence of sub-structures (modularity) [19]	0.301	0.312	0.241

a Excluding isolates

For completeness, we analyzed the networks representing “any” frequency of contact (see Supplementary Information, S1) and found the trends such as overall growth, a dense network with central leadership, and a dip in connectivity in the second wave all to be consistent across both the “regular” and “any” interaction networks.

#### Joint research activities

Beyond measuring interaction frequency, we examined collaboration networks defined by joint engagement in developing new research, publishing manuscripts, and submitting grant applications across the three waves. These networks capture the research activities specifically related to those generating scientific outputs and showed trends consistent with our interaction network findings (Table 2, Fig. 3): substantial growth in both participants and ties, densely connected networks through highly central center leadership, and moderate levels of community structure that remain weakly separated within an overall connected network, varying over time. While we do see minor increases in network span, as indicated by a drop-off in overall density and increased average path length over time, with a process that inherently takes longer, like producing joint research, one would expect to see much more severe impact during the expanding network. This convergence in trends observed across these networks suggests that C-DIAS successfully converted contact into productive research partnerships and that newly added members integrated seamlessly into the collaborative ecosystem.

**Figure 3:**
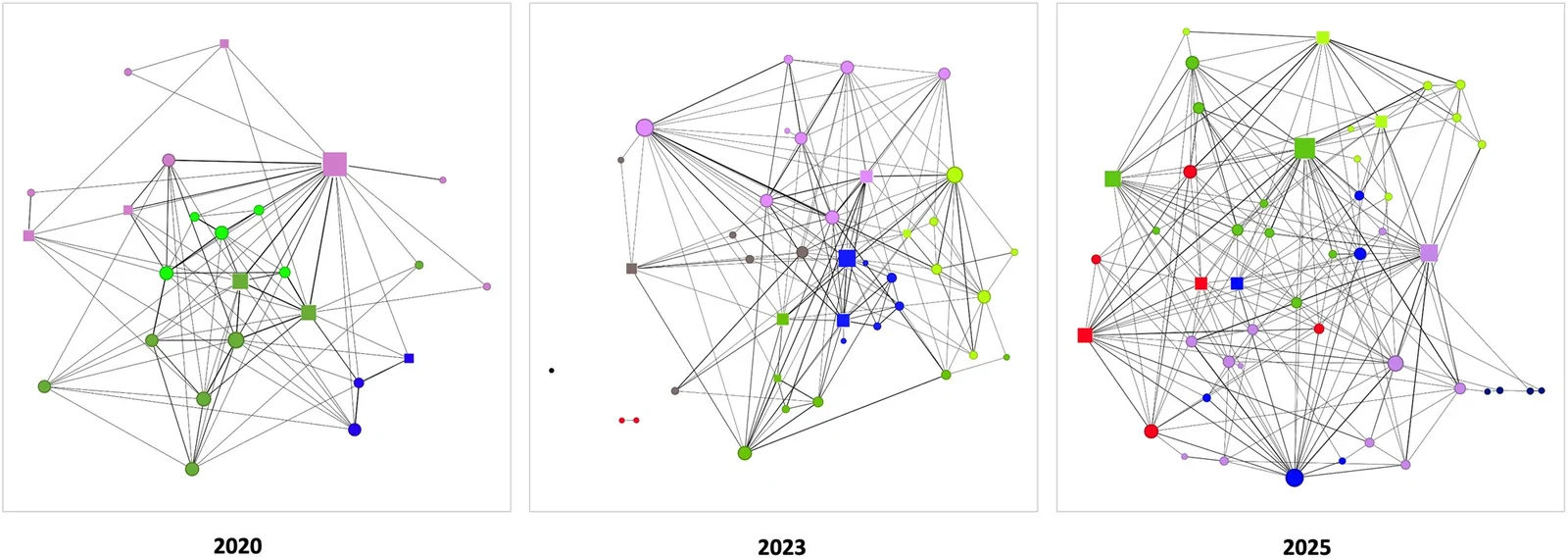
Trends in joint research activities (new research, manuscripts, grants). Figure 3 illustrates the networks of joint research activities within the C-DIAS center for Wave 1 (left), Wave 2 (middle), and Wave 3 (right). Nodes represent individuals (with squares representing Center leadership) and the lines (edges) between them indicate the development of new research, manuscripts, and grants. Node size reflects the relative degree, the number of ties of the individual. Node color in each of these panels captures the community structure of the nodes in that wave specifically.

**Table 2:** Network metrics for joint research activity networks across waves.

	Wave 1	Wave 2	Wave 3
Number of individuals (*N*)	25	39	48
Number of ties (*E*)	91	147	212
Number of ties of the most connected Individual (max degree)	24	22	28
Overall width of the network (diameter)	2	4	4
Average number of steps between individuals (average path length)	1.70	1.99	2.06
Proportion of total ties present (density)	30.3%	19.8%	18.8%
Percentage of neighbors that are connected (average clustering coefficient)	65.8%	47.4%	40.3%
Number of sub-structures (number of communities)	4	7	6
Isolates	0	1	0
Fit with community detection (modularity)	0.235	0.300	0.254

### Bridging disciplinary boundaries

The average number of expertise areas per member increased from 2.0 in Wave 1 to 2.15 in Wave 2 to 2.33 in Wave 3, indicating that new members brought increasingly multidisciplinary profiles. Because expertise areas were coded once and not updated over time, increases in average expertise per member reflect the recruitment of inherently multidisciplinary investigators rather than existing members expanding their domains. This suggests C-DIAS is building capacity for cross-disciplinary work both by training boundary-spanning fellows and by strategically recruiting individuals who already bridge multiple fields.

Growth occurred across all disciplines, not just in specific domains (Table 3). Relative to Wave 1, Addiction health services expertise grew 162%, implementation science 121%, methods and models 118%, and organization and financing of services 100%. This balanced expansion, occurring simultaneously across the full range of competencies, rather than through concentration in any one, indicates increased multidisciplinarity, and is consistent with intentional recruitment across the full range of competencies needed to advance addiction implementation science.

**Table 3:** Expertise in each domain across waves (individuals may have multiple expertise areas).

Domain	Wave 1 (*n* = 25)	Wave 2 (*n* = 40)	Wave 3 (*n* = 49)
Total individuals	25	40 (+60%)	49 (+23% from W2/+96% from W1)
Addiction health services	13	25 (+85%)	34 (+36% from W2/+162% from W1)
Implementation science	19	32 (+68%)	42 (+31% from W2/+121% from W1)
Methods and models	11	19 (+73%)	24 (+26% from W2/+118% from W1)
Organization and financing of services	7	10 (+43%)	14 (+40% from W2/+100% from W1)

### Cross-disciplinary connectivity

Recruiting multidisciplinary individuals is one strategy for breaking silos; fostering connections between individuals with different expertise is another. We examined ties that crossed expertise boundaries; connections between domain experts and non-experts for the “regular” contact network (contacts that occur at least monthly) (Table 4).

**Table 4:** Boundary-spanning ties between domain experts and non-experts, with density (%).

Domain	Wave 1	Wave 2	Wave 3
Addiction health services	36 (23.1%)	52 (13.9%)	100 (19.6%)
Implemention science	9 (7.9%)	25 (9.8%)	52 (17.7%)
Methods and models	29 (18.9%)	43 (10.8%)	71 (11.8%)
Organization and financing of services	22 (17.5%)	48 (16.0%)	106 (21.6%)

Boundary-spanning ties grew substantially in absolute numbers, roughly doubling with each wave. For example, regular cross-disciplinary ties for implementation science increased from 9 to 25 to 52 across waves, while any-frequency ties grew from 21 to 80 to 180. However, raw counts partly reflect network expansion. To account for growth in potential connections, we calculated boundary-spanning density for each domain: the proportion of all possible expert-to-non-expert ties that were present.

Boundary-spanning density was already high in Wave 1, reflecting C-DIAS’s intentional cross-disciplinary design. Following the same pattern observed in overall network metrics, most domains showed a Wave 2 dip in boundary-spanning density followed by recovery or growth in Wave 3. Critically, this pattern held across all four disciplines, demonstrating that the center’s capacity to foster cross-disciplinary connections scaled effectively with network size and was not domain-specific. Implementation science showed particularly strong growth, with boundary-spanning density for any-frequency ties increasing from 18.4% to 31.3% to 61.2%, a pattern suggesting this domain increasingly serves as a bridge connecting other specialties.

### Establishing national reach

To assess C-DIAS’s success as a “center without walls,” we mapped the geographic distribution of member institutions and their interaction patterns. Visual inspection of the three waves (Fig. 4) revealed dramatic geographic expansion. The number of unique institutional locations grew from 10 to 21 to 28 across waves, while connections between locations increased from 23 to 143 to 297. Remarkably, network density remained high (51.1%, 68.1%, 39.3%), indicating that geographic expansion did not dilute interaction intensity. Even as the network spread across greater distances, members maintained frequent contact.

**Figure 4:**
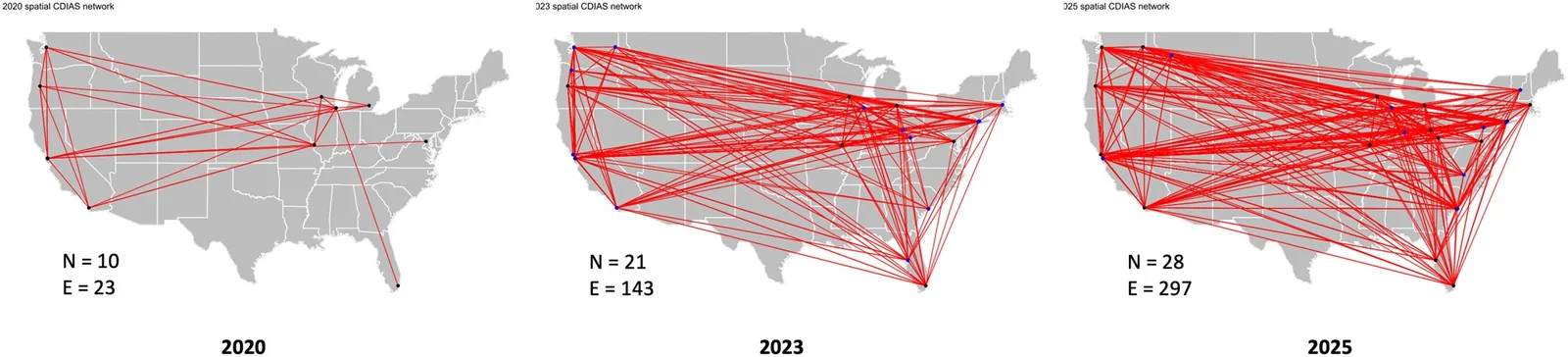
Reach of the C-DIAS network. Figure 4 shows the unique spatial locations of the home institutions of C-DIAS members for Wave 1 (left), Wave 2 (middle), and Wave 3 (right) represented by the dots on the maps, blue dots highlight newly added locations in each of the respective waves. The line across locations captures the presence of interactions across locations that occur at least monthly.

Geographic coverage remained uneven. Institutional locations concentrated heavily on the East Coast, West Coast, and Midwest, with large areas of the Plains and southern United States lacking representation. While C-DIAS successfully built a multi-region network, it has not yet achieved comprehensive national coverage across all geographic areas.

### Building capacity through mentorship

Mentorship networks are critical for developing the next generation of researchers at disciplinary intersections. Our survey captured mentorship ties within the C-DIAS ecosystem across all three waves (Table 5, Fig. 5). Because mentorship is inherently directional, these networks are represented as directed. The mentoring networks reveal three key patterns.

**Figure 5:**
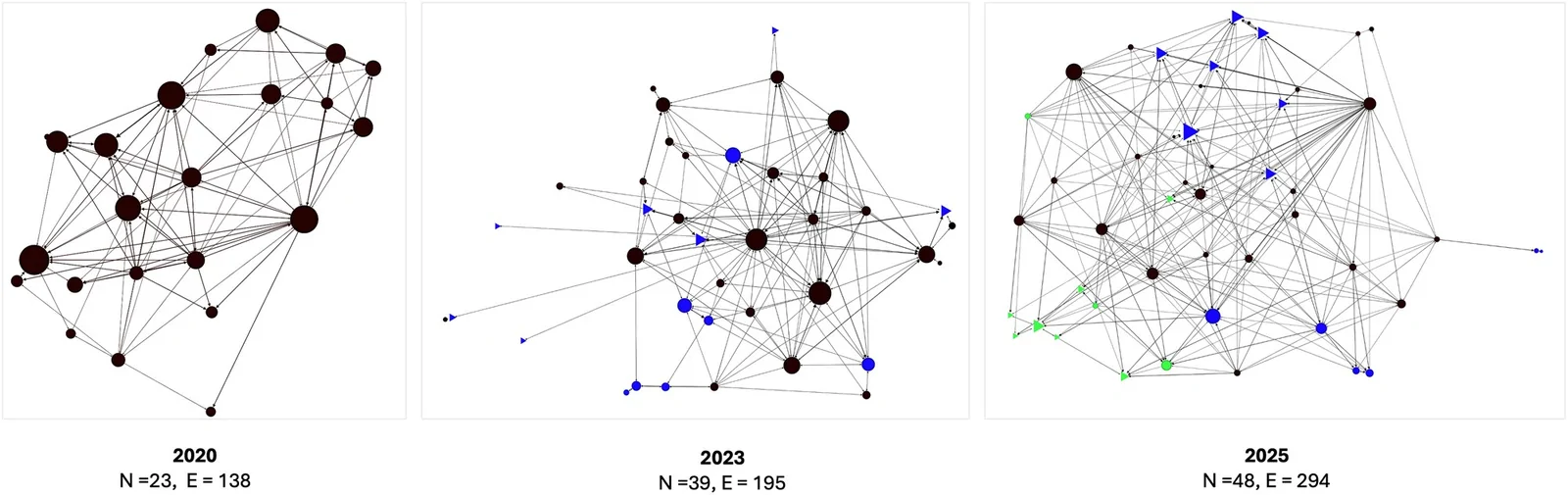
Mentoring networks. Figure 5 illustrates the growth of mentoring networks within C-DIAS. The shape of each node captures that individual’s role, with round nodes representing faculty and triangles representing fellows. The size of the node is proportional to the number of mentoring ties received (in-degree), while node color is based on the wave in which they joined C-DIAS (black = Wave 1, blue = Wave 2, and green = Wave 3).

**Table 5:** Overview of mentoring network attributes.

	Wave 1	Wave 2	Wave 3
Number of individuals active in mentoring	23	39	48
Percentage of individuals active in mentoring	92%	98%	99%
Number of mentors (out-degree > 1)	21	33	46
Number of mentees (in-degree > 1)	22	37	45
Max out-degree	19	29	35
Max in-degree	12	15	16
Total mentoring ties	138	195	294
Bi-directional ties	46 (92 directed)	61 (122 directed)	81 (162 directed)
Number of fellows	0	7	14
Ties to fellows	–	26	117
Ties from fellows	–	2	42

First, participation was near-universal, with 92% (23/25), 98% (39/40), and 99% (48/49) of members engaged in mentorship (as mentor, mentee, or both) in Waves 1, 2, and 3, respectively. These levels indicate that mentorship is a foundational, broadly embedded function within C-DIAS rather than one concentrated among a few highly active mentors. Second, mentorship was predominantly directional rather than reciprocal: in Wave 3, bi-directional ties accounted for 81 of the relationships, with the remaining ties one-directional, consistent with mentorship flowing from senior to junior members. Third, participation and mentorship ties grew substantially over time, and new members were rapidly integrated into the existing structure. Fellows in particular became increasingly embedded, with ties to fellows rising from 26 in Wave 2 to 117 in Wave 3 while ties from fellows grew more modestly (2–42), reflecting their primary role as recipients of mentorship.

As center expansion was driven largely by recruitment of multiple fellow cohorts (triangles in Fig. 5), it is promising to note that over time the embeddedness of the fellows increased, with fellows accounting for increased proportions of mentoring over time [26 ties (∼13%) linked to the seven fellows in Wave 2, and 117 ties (∼40%) to the 14 fellows in Wave 3]. What is more, we observe cohort 1 members continuing to grow the mentorship they received even after cohort 2’s arrival, becoming further embedded in the mentoring network after their initial training period, indicating that mentorship relationships not only persist, but might in fact take longer to fully develop than collaboration ties. This trend in sustained engagement suggests C-DIAS has the potential to provide ongoing support as fellows mature in their careers rather than treating mentorship as time-limited effort.

Diving further into the cohort structure using a radial network diagram (Fig. 6) reveals two additional dynamics. First, newer cohorts, and specifically the fellows, primarily are the recipients of mentorship, second, such mentorship is primarily received from the established core rather than from individuals within their own or prior cohorts. This suggests mentorship flows primarily from senior to junior members rather than laterally across cohorts, and as a result the network is likely to exhibit distinct generational layers.

**Figure 6:**
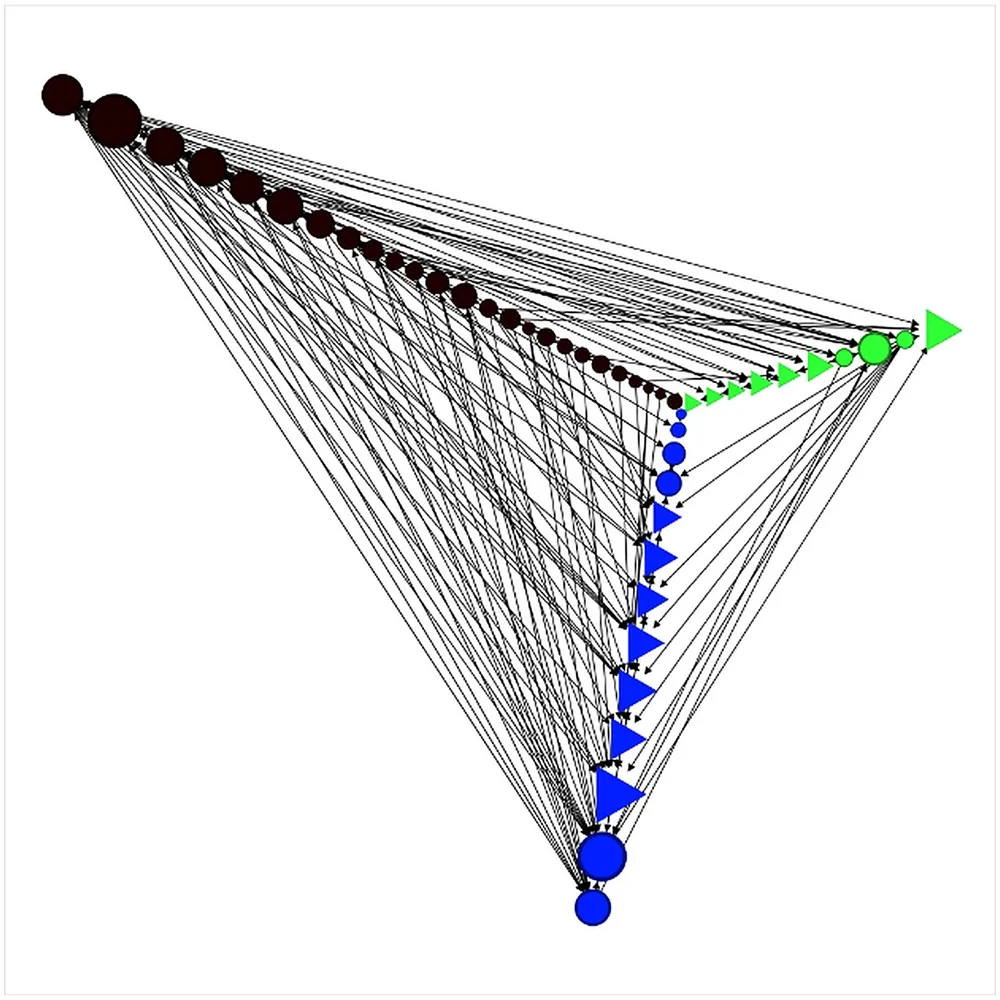
Radial view of cross-cohort mentoring. Mentoring networks within C-DIAS (Wave 3 data). To highlight the connections between cohorts this projection places each cohort of individuals on its own radial. Fellows are represented as triangles. Individuals are colored based on the wave in which they entered the C-DIAS network (black = Wave 1, blue = Wave 2, and green = Wave 3), and their size is proportional to the number of mentee ties (in-degree).

## Discussion

Longitudinal SNA was used to evaluate the collaborative evolution of the NIH funded C-DIAS. Our analysis revealed that C-DIAS has successfully formed and grown a network of addiction health services researchers with implementation science expertise.

### Growth of interaction and collaboration networks

C-DIAS has created the collaborative infrastructure necessary to bridge the research-to-practice chasm by systematically integrating implementation science and addiction health services expertise. Boundary-spanning ties connecting these disciplines grew from 21 in Wave 1–180 in Wave 3. This structural integration created the collaborative ecosystem needed to tackle complex implementation challenges. For example, expanding MOUD access requires not only knowledge of buprenorphine pharmacology and AT protocols but also understanding of how to overcome organizational barriers, modify clinical workflows, address provider stigma, and sustain practice change. Neither discipline alone possesses the full toolkit; synergy emerges at their intersection.

Both interaction and collaboration networks grew substantially while maintaining remarkably high connectivity. Network density remained above 58% across all waves for any-frequency interactions and above 19% for regular monthly contact, even as the network doubled in size. This sustained engagement density reflects the frequent, meaningful exchange between addiction and implementation science experts necessary for true integration rather than superficial collaboration [20]. The Wave 2 dip in connectivity metrics followed by Wave 3 recovery appears consistently across measures, suggesting natural growing pains as C-DIAS transitioned from a small founding group into scaled operations. Wave 2 coincided with rapid expansion as multiple fellow cohorts joined while launching multiple implementation projects. By Wave 3, connectivity rebounded through formalized working groups, established communication rhythms, and norms for distributed collaboration, demonstrating organizational learning and suggesting that temporary decreases during rapid growth should be addressed through intentional infrastructure development rather than interpreted as program failure.

### Integrating disciplinary silos

Breaking down the disciplinary silos traditionally separating addiction health services research, implementation science, methods and models, and organization and financing of services, and building research capacity at the intersection of these disciplines is a primary goal of the C-DIAS center. With cross-disciplinary connections increasing proportionally with network growth, the expansion of the C-DIAS network over time inherently contributed to an expansion of the cross-disciplinary capacity within the field. What is more, our analysis documents that capacity was built not only through fostering connections between individuals with different expertise domains, but also by recruiting investigators with inherently multidisciplinary profiles. The average number of expertise areas per member within C-DIAS increased from 2.0 to 2.33 across waves, with balanced growth across all four disciplines rather than concentration in specific domains. This highlights the intentional recruitment of boundary-spanning investigators, in addition to connecting them to the existing network to increase their chances of success. Because expertise was coded once and not updated over time, this increase is attributable to who was recruited rather than to existing members broadening their domains: members added between Waves 1 and 2 carried, on average, 2.4 expertise areas each, and those added between Waves 2 and 3 carried 3.13, both above the standing network average (2.0–2.33). New members were thus, on balance, more multidisciplinary than the existing network, not simply additional single-discipline nodes.

### Building capacity through mentorship

Mentorship networks achieved near-universal participation (99% by Wave 3), creating pathways for developing the next generation of investigators positioned as addiction health services implementation scientists. This capacity-building function addresses a critical workforce gap. As NIH’s HEAL Initiative emphasizes, accelerating translation of research to practice requires investigators trained in implementation research specifically for substance use prevention and treatment [21], yet few training programs systematically develop both skill sets. Mentorship is embedded throughout C-DIAS rather than concentrated among a few highly active individuals. The hierarchical structure, with fellows receiving mentorship primarily from established faculty rather than from each other, creates systematic pathways for developing boundary-spanning competencies. Early career investigators receive exposure to both implementation science frameworks and addiction health services expertise.

We note that the limited cross-cohort mentoring between earlier and later fellow cohorts potentially represents a missed opportunity. Peer mentorship across cohorts could accelerate integration by creating lateral knowledge transfer pathways. Earlier cohorts could provide developmentally proximate guidance to newer fellows. Structured peer mentoring might also reinforce earlier cohorts’ integration as they transition from primarily mentee roles toward emerging mentorship capacity themselves.

The persistence of mentorship relationships beyond initial training periods highlights C-DIAS’ belief in professional development as a longitudinal process rather than time-limited training. This extended support may be particularly important for implementation science, where successful independent research requires not only methodological expertise but also skills in stakeholder engagement, policy navigation, and building sustainable partnerships; competencies that develop gradually through sustained mentored experiences.

### Establishing national reach

C-DIAS was intentionally designed as a “center without walls”; a geographically distributed network intended to serve as a national resource. Our analysis documents both the promise and limitations of this model. The number of unique institutional locations grew dramatically from 10 to 28 across waves, while connections between locations increased from 23 to 297. Remarkably, network density remained high (39%–68%), indicating that geographic expansion did not dilute interaction intensity.

While expansion was evident, geographic coverage remained uneven, with institutional locations concentrated heavily on the East Coast, West Coast, and Midwest. Large areas of the Plains and southern United States lack representation. While C-DIAS successfully built a multi-region network, it has not yet achieved comprehensive national coverage. Future expansion efforts might strategically target underrepresented geographic areas, particularly if these regions also face limited access to implementation science expertise, creating dual value through both network expansion and more equitable distribution of scientific capacity.

The success of this distributed model challenges assumptions that implementation work requires geographic concentration. However, it required deliberate infrastructure: regular virtual meetings, collaborative workspaces, project management systems, and shared commitment to a translational mission larger than any individual project. The viability of distributed networks has implications for AT, where implementation challenges vary dramatically across urban, rural, and frontier settings.

### Limitations

As a single-center study, our findings may reflect characteristics unique to C-DIAS’s structure, institutional context, or leadership. Comparative studies across multiple research or collaboration centers are needed to identify generalizable principles of network development. Furthermore, our reliance on self-reported collaboration data introduces potential bias, though the consistency of findings across waves and network metrics, and the nearly 100% completion rate, suggests robust patterns. While we measured network structure, we did not directly assess outcomes such as the quality of collaborations, the impact of resulting research, or the extent to which C-DIAS activities accelerated translation in addiction services. It is also important to note that these results only reflect the first few years of the center. Future work should look beyond those early years and link network characteristics to productivity and impact. Finally, our definition of network boundaries, based on affiliation with C-DIAS, necessarily excluded collaborations that occurred outside the center’s formal structure, potentially underestimating members’ total collaborative activity.

## Conclusions

The addiction health services field faces particularly acute implementation challenges, with evidence-based treatments reaching only a small fraction of individuals who could benefit. These implementation failures reflect not only scientific gaps but also fragmentation across treatment systems, policy domains, and research disciplines. C-DIAS’s demonstrated success in building collaborative infrastructure across these traditionally siloed domains suggests that center-based approaches may be particularly valuable for fields where implementation problems are inherently complex and multifaceted.

Our findings suggest that collaborative or research centers can function as translational accelerators through several mechanisms. First, they create sustained opportunities for repeated interaction, allowing trust and shared understanding to develop across disciplinary boundaries. Each successful joint project becomes a foundation for future partnerships, reducing the transaction costs of initiating new translational efforts. This aligns closely with the vision of “practice-based translational behavior change research,” [22] wherein research is embedded within clinical and public health settings from inception. The involvement of health system partners, clinicians, and community stakeholders in the collaborative network ensures that research questions emerge from practice needs rather than solely from theoretical or methodological interests. The boundary-spanning ties documented in our analysis represent more than statistical patterns; they reflect ongoing relationships through which tacit knowledge about implementation contexts flows back into intervention development. When implementation scientists collaborate with addiction researchers from the earliest stages of grant development, questions of feasibility, acceptability, and sustainability shape study design alongside questions of efficacy. This integration transforms implementation from an afterthought into a core scientific objective, fundamentally altering the types of evidence generated and their relevance to practice settings. Second, cross-disciplinary centers provide structured mechanisms that lower barriers for those wanting to pursue multi-disciplinary research. Training the next generation of scientists in transdisciplinary collaboration ensures that implementation considerations become integrated into behavioral medicine research from the earliest stages rather than appended post-hoc. For policymakers and funding agencies, these findings carry important implications. The NIH has increasingly emphasized team science and implementation research, yet funding mechanisms often remain structured around individual principal investigators and single-institution projects. Our data suggest that sustained investment in collaborative infrastructure, including core resources for centers, support for network-building activities, and incentives for cross-institutional partnerships, may yield disproportionate returns by accelerating the entire discovery-to-delivery continuum.

By intentionally cultivating collaborative networks that span disciplinary, institutional, and geographic boundaries, research and collaborative centers create conditions for research that is simultaneously rigorous and relevant, creating collaborative infrastructure positioned to accelerate evidence translation during ongoing public health crises. Over three years, C-DIAS established a highly connected, nationally distributed network characterized by dense cross-disciplinary interaction, productive collaboration, and systematic capacity-building. This analysis provides evidence of center-led collaboration and offers insights into the processes by which C-DIAS broke down disciplinary silos to advance addiction health services implementation science.

## Supplementary Material

ibag074_Supplementary_Data

## Data Availability

De-identified data from this study may be available by emailing the corresponding author.
