# Supplementary material for: Building bridges, breaking silos: a social network analysis of collaboration in an implementation science center for addiction health services
Source: Transl Behav Med. 2026 Oct 7;16(1):ibag074. doi: 10.1093/tbm/ibag074 (PMC13644531; doi:10.1093/tbm/ibag074)
Supplement: Supplementary file 1 [file ibag074_supplementary_data.docx]

**Supplementary Information (S1)**

*Figure S1 displays networks of interactions within the C-DIAS center for Wave 1 (left), Wave 2 (middle), and Wave 3 (right). Circles (nodes) represent individuals and the lines (edges) indicate interactions (disregarding the frequency). Colors represent the community the nodes belong to.*
